# No link between handedness and spatial navigation: evidence from over 400 000 participants in 41 countries

**DOI:** 10.1098/rspb.2023.1514

**Published:** 2023-10-11

**Authors:** P. Fernandez-Velasco, A. Coutrot, H. Oloye, J. M. Wiener, R. C. Dalton, C. Holscher, E. Manley, M. Hornberger, H. J. Spiers

**Affiliations:** ^1^ Institute of Behavioural Neuroscience, Department of Experimental Psychology, Division of Psychology and Language Sciences, University College London, London, UK; ^2^ Institute of Cognitive Neuroscience, Division of Psychology and Language Sciences, University College London, London, UK; ^3^ Centre of Medical Imaging Computing, Department of Computer Science, Faculty of Engineering Sciences, University College London, London, UK; ^4^ Centre for Advanced Spatial Analysis, University College London, London, UK; ^5^ Department of Philosophy, Trinity College Dublin, Dublin, Ireland; ^6^ Department of Philosophy, University of York, York, UK; ^7^ LIRIS, CNRS, University of Lyon, Lyon, France; ^8^ Department of Psychology, Ageing and Dementia Research Centre, Bournemouth University, Poole, UK; ^9^ Department of Architecture and Built Environment, Northumbria University, Newcastle upon Tyne, UK; ^10^ ETH Zurich, Swiss Federal Institute of Technology, Zurich, Switzerland; ^11^ School of Geography, University of Leeds, Leeds, UK; ^12^ Norwich Medical School, University of East Anglia, Norwich, UK

**Keywords:** handednesses, navigation, cross-cultural, education, spatial cognition

## Abstract

There is an active debate concerning the association of handedness and spatial ability. Past studies used small sample sizes. Determining the effect of handedness on spatial ability requires a large, cross-cultural sample of participants and a navigation task with real-world validity. Here, we overcome these challenges via the mobile app Sea Hero Quest. We analysed the navigation performance from 422 772 participants from 41 countries and found no reliable evidence for any difference in spatial ability between left- and right-handers across all countries. A small but growing gap in performance appears for participants over 64 years old, with left-handers outperforming right-handers. Further analysis, however, suggests that this gap is most likely due to selection bias. Overall, our study clarifies the factors associated with spatial ability and shows that left-handedness is not associated with either a benefit or a deficit in spatial ability.

## 1. Introduction

The impact of handedness on cognition is a question of longstanding interest across several domains [1–6]. One of these domains concerns spatial cognition. In an experiment by Piper *et al.* [7], 287 volunteers undertook the navigation test Memory Island, designed after the Morris water maze. Participants found themselves in a virtual island. First, they had to navigate between locations (e.g. a sculpture, a seagull, etc.) marked with a big flag in order to memorize them. Then, the flag disappeared, locations became hidden and participants had to find them based on spatial memory. What Piper and colleagues found, contrary to their expectations, was that left-handers were better than right-handers at this navigation task: they were able to find the target locations while covering shorter distances. This result aligns with previous work by Annett [8], who found that left-handers enjoy a cognitive advantage for spatial tasks (*n* = 428). Annett employed a spatial ability test in which participants had to do a mental paper folding task designed to measure spatial visualization. More recently, left-handers outperformed right-handers on spatial ability in Mazes-Tracing, Hidden Figures and Cube Perfectives tests ([9], *n* = 225). An explanatory analysis of reports of difficulty in spatial behaviour points in a similar direction: right-handedness was associated with more perceived difficulty in judging spatial relations while driving, overlaying objects and moving in relation to other objects in nearby space [10]. Finally, it is well known that in professional sports that require rapid and accurate responses, athletes with a left preference (e.g. holding a bat with their left hand) seem over-represented, something that would also indicate a left-handed advantage in spatial abilities [11].

A possible hypothesis explaining the purported advantage of left-handedness in spatial tasks relates to brain lateralization. As is the case with verbal ability, for most humans, the neural underpinning of spatial ability has been argued to be lateralized to either of the two brain hemispheres [12]. Cerebral lateralization, as the phenomenon is known, correlates (although not strongly) with hand preference. For instance, for language processing, up to 95% of right-handed people show left-cerebral dominance, in contrast with 75% of left-handed individuals [13]. A meta-analysis focusing on spatial tasks, found that these are largely controlled by the right hemisphere [14], which aligns with the idea that spatial functions are located in the right hemisphere of the brain [15–17]. Interestingly, the meta-analysis by Vogel and colleagues [14] also found that left-handers were lateralized differently from right-handers. Previous studies have shown that left-handers outperform right-handers in executive tasks that typically engage the right hemisphere [18]. By analogy, a possibility is that left-handers outperform right-handers in spatial tasks that typically engage the right hemisphere.

However, the link between spatial ability and handedness is a matter of controversy and mixed results. Mellet *et al.* [19] (*n* = 436) employed a battery of tests of spatial ability (mental rotation test, Corsi block test, a three-dimensional maze and the Raven matrix for non-verbal reasoning) and found no effect linking left-handedness and spatial ability. Several studies ([20], *n* = 359; [21], *n* = 89) found right-handers to be superior to left-handers (using the Stafford identical block test and mental rotation tests, respectively). Going back to the increased prevalence of left-handedness is elite athletes, it is important to note that the left preference for sport tasks is not necessarily an indicator of left-handedness [22]. Moreover, a sport-by-sport analysis found that effects for a left-handedness advantage are slight and disappear for sports in which there is no strategic left-handed advantage (football goalkeepers), so the most parsimonious explanation for the purported over-representation of left-handers in sport is that any left-handed advantage reflects the nature of the game rather than a general advantage in spatial ability [23].

Within laterality research, there is a tendency for meta-analysis to resolve issues surrounding mixed results [24]. In this case, a meta-analysis found that right-handers slightly outperformed others in spatial tasks [25]. A possible reason for a right-handed advantage in spatial tasks also involves brain lateralization. Early in the history of handedness studies, Levy proposed an advantage of right-handers in spatial tasks [16]. She reasoned that left-handers would have a higher right-hemispheric language function, and that consequentially, fewer neural resources would be devoted to spatial functions. Here again, the issue comes down to lateralization. What seems clear is that the differences in lateralization patterns of cognitive functions may underlie a benefit or deficit pertaining to spatial ability in left-handers. Finally, an important element is that hemispheric lateralization is likely to be graded and emerge dynamically over the course of development [26], so that the lateralization of one function might be dependent on the lateralization of another function, an idea known as complementary hemispheric specialization. The complementary specialization in the two hemispheres resulting from increased lateralization in right-handers might increase overall computational efficiency because it avoids unnecessary duplication of critical neural tissue (as suggested by Powell *et al.* [27]), which might be key in complex functions such as spatial cognition.

The first question, however, is to assess whether such a handedness-related benefit or deficit exists. This is a complicated question. In the meta-analysis by Somers and colleagues [25], the majority of the studies analysed tackled this question with few participants. In fact, the effect failed to reach significance when a single, large study was excluded from the meta-analysis ([28], *n* = 210 916). This suggests that a robust association of hand preference with spatial cognition necessitates a large sample. This is especially pressing when one considers the low effect size for the association of handedness and spatial ability in the meta-analysis by Somers and colleagues ([25], Hedges' *g* = −0.14), as well as the high heterogeneity (I2 = 82). Part of the issue is that there are many differing tests of spatial abilities. When the meta-analysis looked only at mental rotation tasks, they found a similar effect size (Hedges’ *g* = −0.13) but a moderate heterogeneity index (I2 = 59). Crucial here is that most studies have focused on small-scale spatial tasks (e.g. mental rotation; [29]), rather than on large-scale spatial cognition (e.g. navigation). While performance in large- and small-scale spatial abilities is significantly correlated, the correlation is low to moderate [30–32]. The lack of a strong correlation between performance in small- and large-scale spatial tasks indicates that, while they have some overlap, they also make different demands on cognition [31,33]. An important difference between wayfinding and other spatial tasks is that wayfinding poses specific demands on planning and inhibition [34].

Another source of complexity when studying the link between handedness and large-scale navigation comes from the fact that cultural differences have a significant impact on both. Differences in nationality and culture are associated with variation in spatial navigation ability [35–37]. Walkowiak *et al.* [38] analysed the relationship between self-estimates of navigation ability and performance in a navigation task and found that cultural clusters of countries tend to be similar in how they self-rate ability relative to their actual performance and that cultural dimensions such as masculinity (i.e. positive attitudes to male stereotyped roles) affected the gap between self-rated ability and actual performance. Like navigation ability, hand-preference distribution also varies widely between countries, probably due to different cultural pressures [39–41]. A recent meta-analysis found that participants of European ancestry had a much higher prevalence of left-handedness (11.12%) compared with participants of sub-Saharan African ancestry (7.71%), or of East Asian ancestry (5.69%) [42]. Together, the cross-cultural variation in both spatial ability and handedness complicates studying the association between the two.

Importantly, both handedness and navigation ability vary not only across, but also within populations [37,43]. Previous demographic studies have shown a higher proportion of males use their left hand, and studies of navigation ability suggest a male advantage in spatial tasks [35,44,45]. Part of this difference might relate to handedness. One possibility is that there is an interaction between handedness and gender when it comes to spatial ability. For instance, a study found that left-handed males had higher spatial scores than right-handed males, whereas left-handed females had lower spatial scores than right-handed females ([46], *n* = 879). Regarding brain asymmetries, the meta-analysis of spatial ability and cerebral lateralization mentioned earlier showed that females are less generally lateralized for spatial tasks than males [14]. Hand preference also varies depending on age, perhaps reflecting a change in tolerance towards left-handers over time, as older people are more likely to have been forced to switch handedness [2,39].

A further motivation for clarifying whether handedness is associated with navigation performance concerns the design of neuroimaging studies. If there are differences in spatial ability associated with hand preference, this would have consequences for experimental design: left-handers are routinely excluded from brain imaging studies [14,47,48]. However, if handedness is associated with spatial navigation ability, the exclusion of 10% of the population potentially leaves an aspect of spatial cognition underexplored.

The challenge in addressing the link between handedness and spatial ability is twofold. First, it is difficult to test for large-scale spatial ability (i.e. navigation) with a method that is predictive of real-world performance. Second, the impact of culture on both handedness and spatial ability, compounded with the potentially small effects, means that tackling the question would require a large sample size. Even when testing the link between handedness and small-scale spatial ability, the majority of existing studies have had relatively small sample sizes. This means factors such as age and gender might not be adequately accounted for, which potentially explains the divergence in results across studies [10].

Here, we overcame past limitations by using Sea Hero Quest (SHQ), a gamified navigation task. The ecological validity of our spatial ability metric was tested in a previous experiment in which we compared participants' performance in a subset of wayfinding levels with performance in a real-world wayfinding task in the area of Covent Garden in London and found a significant correlation (*r* = 0.46) between the distance participants travelled in the video game (in pixels) and in the real-world street network (in metres, measured by a GPS device), a result that was replicated with another group of participants in the area of Montparnasse in Paris (*r* = 0.57) ([49], *n* = 49, 25 males, aged 18–30 years old). These results are consistent with existing studies showing that spatial navigation assessment in both desktop and immersive environments transferred well to the real world [50–53].

Using SHQ, we are able to test a large, diverse sample of individuals from 41 countries worldwide. The main aim of the study was to establish the association between handedness and spatial ability. Additionally, we wanted to determine the distribution of hand preference across nations, clarify how it connects to socio-demographic factors such as age, gender and education, and explore if those socio-demographic factors mediate the relationship between handedness and spatial ability.

## 2. Methods

### (a) Participants

#### (i) Data collection

Fulfilling the aim of collecting data from millions of participants worldwide required an optimal data collection strategy and advertising of the Sea Hero Quest app. Data were recorded to the participant's device locally and sent encrypted to a secure server. Participants downloaded the game Sea Hero Quest as an app. There was no financial compensation for participation, and the motivation was only to contribute to research by playing. Saatchi and Saatchi Ltd. were key in the advertisement for the project, developing several film and animation adverts about the study. The game also allowed players to share progress via Facebook and Twitter. Deutsche Telekom specifically advertised the game to its millions of customers, and Alzheimer's Research UK promoted it to its supporters. The game was advertised through social media and through a press release, and it received extensive media coverage. This advertisement effort led to Sea Hero Quest becoming the most downloaded app on the Apple App store for a short period. This way, we were able to recruit over 4 million participants from every country in the world (see [36] for more details).

#### (ii) Demographic analysis

Of Sea Hero Quest, 3 881 449 people played at least one level. Participants that had not entered all of their demographics were excluded from this study, as were participants who were over 70 years old, a group with strong selection bias, which has previously been shown to result in increased performance [35]. Only countries with at least 1000 players were included in our sample. This resulted in an analytic sample of 749 037 participants (390 732 males) from 58 countries with a mean age = 38.64 (s.d. = 14.56); 535 325 received tertiary education (university or college), 213 389 received secondary education or lower; 74 444 were left-handers (9.94%).

#### (iii) Spatial ability analysis

Starting from the same analytic sample as the demographic analysis, only participants who had completed the first 11 levels of the game were included in the analysis. This ensured a reliable estimate of spatial navigation ability in our analytic sample. We chose the cut-off at level 11 because it was a good trade-off between sample size and data robustness (the more levels we include, the more robust the data, but the smaller the sample size). This selection resulted in an analytic sample of 422 772 participants (226 087 males) from 41 countries with a mean age = 37.81 (s.d. = 14.17); 42 232 were left-handers (9.99%).

### (b) Materials

**Figure 1.**
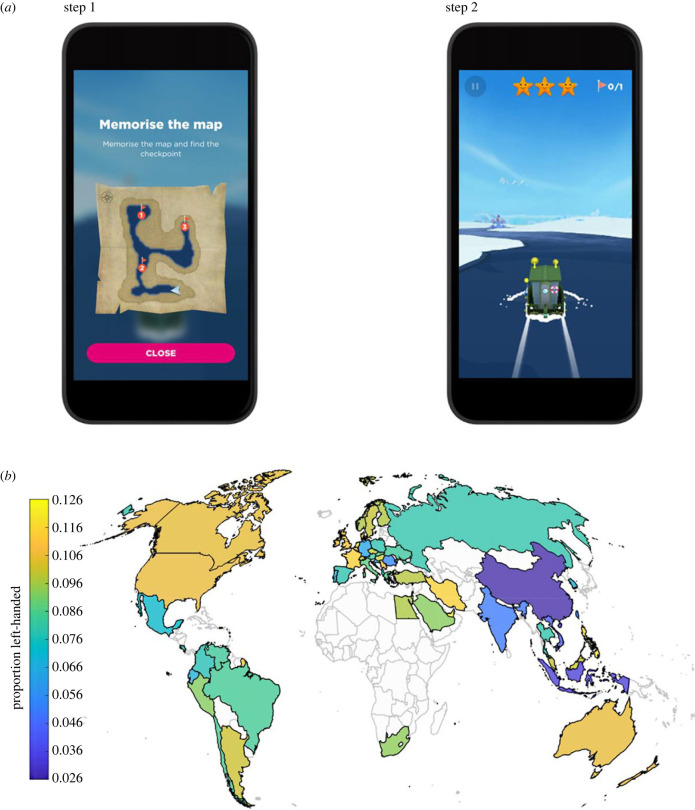
(*a*) Wayfinding task in the Sea Hero Quest app. Images show example screenshots from the game as they would appear on a mobile device. Step 1 involved viewing a map of the environment indicating the layout, current location (arrow) and checkpoints to navigate to in a given order. In the example above (level 11) the three checkpoints were used. Across game levels, these varied from 1 to 5. After viewing the map, participants pressed the ‘close’ icon and the task transition to step 2. In step 2, participants tapped the left and right of the boat to steer it to the checkpoints and could swipe up to speed up or swipe down to slow the boat. (*b*) Map of left-handedness rate across countries.

Sea Hero Quest is a mobile video game that measures spatial navigation ability [35,36]. In wayfinding levels (45 levels out of a total of 75 levels), Sea Hero Quest asks participants to view a map featuring both their current position and their goal locations (figure 1*a*). Participants are then asked to navigate a boat as quickly as possible towards goal locations in a specified order (figure 1*b*). We selected a subset of four wayfinding levels of increasing but moderate difficulty appearing quite early in the game (levels 6, 7, 8 and 11)*,* alongside two training levels (levels 1 and 2). We made the decision not to include levels that measured path integration, because the performance parameter was a categorical 1–3 score with low sensitivity and limited variation in response [49]. Consent for the study was provided by the University College London (UCL) ethics board, and informed consent was provided within the app.

Participants indicated handedness by selecting a hand on either the left or right side of the screen before they began the game. Participants were asked: ‘What hand do you write with (dominantly)?’ For analysis, age was from 19 to 69, gender had two classes (males, females), handedness had two classes (left, right) and education had two classes (up to secondary, tertiary).

Participant spatial ability was measured using the Euclidean distance travelled in each wayfinding level. The coordinates of their trajectories were sampled at Fs = 2 Hz. In order to account for videogame skill (i.e. at controlling the boat using a smartphone), we normalized the trajectory length in each level by the sum of the distances travelled over tutorial levels 1 and 2*.* These tutorial levels did not require any spatial ability and were designed to measure participants' ability to control the virtual boat. This resulted in normalized trajectory lengths for the four wayfinding levels under study (6, 7, 8 and 11). Finally, we conducted a principal component analysis over the normalized trajectory lengths of the four wayfinding levels included in the analysis. We defined the wayfinding performance metric (WF_perf) as the first component of this principal component analysis (as in [35]). Therefore, for each participant under study, we had a corresponding wayfinding performance metric, which was our measure of the participant's spatial navigation ability.

## 3. Results

### (a) Demographics

We fit a multi-level logistic regression model with handedness as the response variable, age, gender and education as fixed effects, and country as random effect (handedness approx. age + gender + education + (1|country)). All dependent variables had a significant effect on handedness: age (*F*_1,748708_ = 464.45, *p* < 0.001), gender (*F*_1,748708_ = 925.56, *p* < 0.001) and education (*F*_3,748708_ = 48.435, *p* < 0.001). The standard deviation of the country random effect was 0.34, 95% CI = [0.28, 0.41]. The variance partitioning coefficient (VPC), i.e. the proportion of observed variation in handedness that is attributable to the effect of clustering by country, is 3.61%.

**Figure 2.**
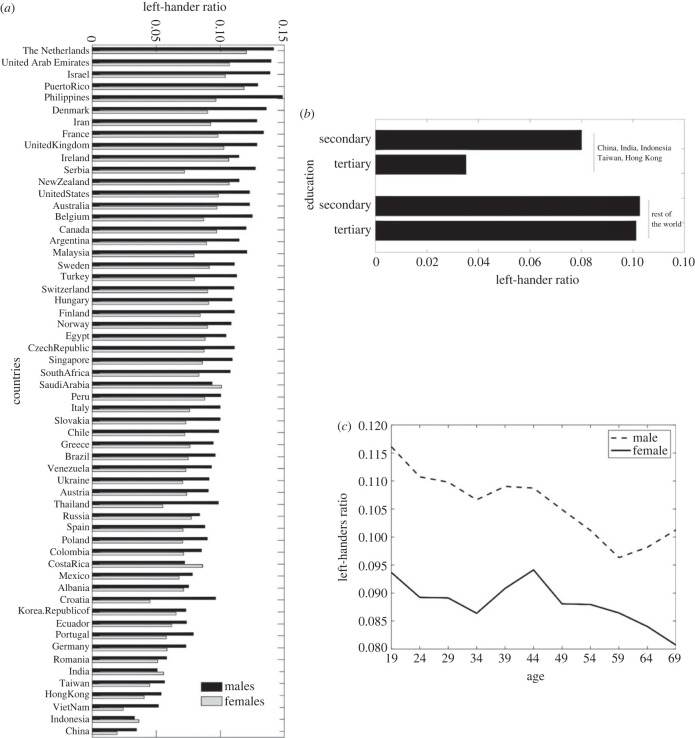
(*a*) Left-handers ratio across countries, for males and females. (*b*) Left-handers ratio in participants with tertiary education and with secondary education or lower. The first two bars correspond to participants from China, India, Indonesia, Taiwan and Hong Kong, which have the lowest left-handers ratio. The second two bars correspond to participants from other countries. (*c*) Left-handers ratio across age and gender.

The incidence of left-handedness in our sample was 9.94% and was smaller in females (8.95%) than in males (10.85%). It decreased with age (10.76% at 19 years old versus 8.68% at 70 years old, figure 2*c*) and with level of education (9.82% in participants with tertiary education, 10.25% with secondary education or lower, figure 2*b*). Looking across countries, the gender effect is fairly consistent, with the exceptions of India, Indonesia, Costa Rica and Saudi Arabia, where females are slightly more likely to use their left hand than males, figure 2*a*. The Netherlands has the highest left-handers rate (12.95% left-handers), while China has the lowest (2.64% left-handers). The increase in left-handedness in participants with lower levels of education was mainly driven by China, Indonesia, India, Taiwan and Hong Kong (*N* = 26 223), where there is a lower rate of left-handers and where the education effect was much stronger than in the other included countries (*N* = 722 814, see figure 2*b*). In China, Indonesia, India, Taiwan and Hong Kong, the average difference in left-hander ratio between participants with and without tertiary education was 4.49% (*χ*^2^ = 96.74, *p* < 0.001), while in the rest of the world it was 0.15% (*χ*^2^ = 3.54, *p* = 0.06).

### (b) Spatial ability

**Figure 3.**
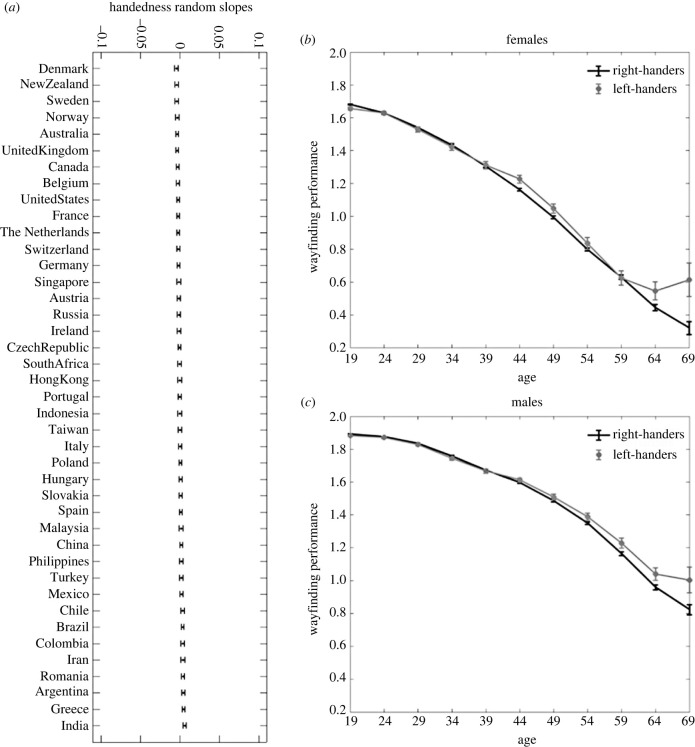
Navigation performance compared across countries, age and gender. (*a*) Effect of handedness across countries, ordered by effect size. We fit a linear mixed model for wayfinding performance, with fixed effects for age, gender, education and handedness, and random effect for country. We plot the handedness slopes for each country. To visually compare the magnitude of the variation of the handedness effect across countries to the variation of the effect of gender across countries, the *x*-axis limits are set to the maximum values of the gender slopes across countries. (*b–c*) Effect of handedness on wayfinding performance across age for males and females. The wayfinding performance has been averaged within 5-year time windows. We consider the apparent increase in performance in later life to be derived from a selection bias and plot data up to the age of 69, figure 4 for our analysis of the selection bias. Error bars correspond to 95% confidence intervals.

We fit a multi-level linear model with wayfinding performance as the response variable, handedness, age, gender and education as fixed effects, with random slopes for handedness clustered by country (WF_perf approx. age + gender + education + handedness + (handedness|country)). Age (*F*_1,422767_ = 69094, *p* < 0.001), gender (*F*_1,422767_ = 23308, *p* < 0.001) and education (*F*_3,422767_ = 514.77, *p* < 0.001) had a significant effect on wayfinding performance. By contrast, handedness did not have a significant effect (*F*_1,422767_ = 1.72, *p* = 0.19). We measured the effect size of handedness on wayfinding performance with Hedges' *g*. Overall, *g* = 0.045, 95% CI = [0.035, 0.055] (in females *g* = 0.024, 95% CI = [0.008, 0.039], in males *g* = 0.027, 95% CI = [0.014, 0.04]), positive values corresponding to better performance in left-handers. As a point of comparison, for gender, *g* = 0.44, 95% CI = [0.43, 0.45], positive values corresponding to better performance in males. The standard deviation of the handedness effect across countries was 2.9 × 10^−3^, 95% CI = [1.3 × 10^−4^, 6.4 × 10^−2^], which was very small compared with the residual standard deviation (0.80, 95% CI = [0.79, 0.80] and suggests that the differences of the handedness effect size between countries are negligible. This is illustrated in figure 3*a* which shows the handedness slopes for each country. We see that there is very little variation across countries. To visually compare the magnitude of the effect of handedness with the effect of gender, the *x*-axis limits are set to the maximum values of the gender slopes across countries (−0.1,0.1).

**Figure 4.**
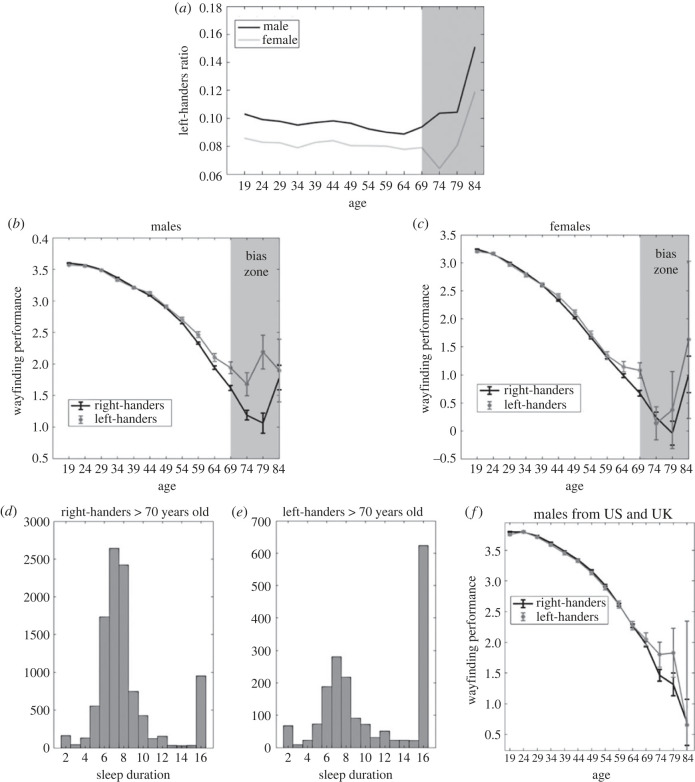
Analysis of selection bias. (*a*) Left-handers ratio across age, for males and females. The ratio changes sharply after 70 years old. The area in question is shadowed in the figure and marked as the ‘bias zone’. (*b*,*c*) Effect of handedness on wayfinding performance across age for males and females. The wayfinding performance has been averaged within 5-year time windows. Error bars correspond to 95% confidence intervals. (*d*,*e*) Distribution of sleep duration for over 70 years old right-handers and left-handers. (*f*) Effect of handedness on wayfinding performance across age for males from the US and the UK.

Figure 3*b*,*c* shows the effect of handedness on wayfinding performance across age for males and females, respectively. Across the life course, there is no difference in performance between left- and right-handers for males and females. A small but growing gap in performance appears for participants over 64 years old, with left-handers outperforming right-handers (note that wayfinding performance has been averaged within 5-year time windows). This gap is most likely due to selection bias. Past research on cognitive ageing predicts gradual declines in performance with age, but rather we observed an inflection with improving performance after approximately 70 years old [35,54,55]. Figure 4 provides a further analysis of the issue. Figure 4*a* shows the left-handers ratio across age, for males and females. It is evident that after 70 years old, the ratio increases sharply. We deemed the area of the sharp increase in the left-handers ratio a ‘bias zone’. There seems to be a selection bias for those participants, since it is unlikely that such a sharp increase would occur for actual handedness. This is further supported by the analysis of sleep patterns in figure 4*d*, which compares the distribution of sleep duration for over 70-year-old right-handers and left-handers. Those reporting to be left-handed show a substantive increase in reported sleep duration of over 16 h a day for the group over 70, which is a pattern not predicted from laboratory studies [55]. It is not clear why this selection bias occurs in this manner. Another possibility is that some participants misrepresent their demographics, selecting ages and sleep duration on the higher ends of the options given to them, and selecting handedness inaccurately. The higher spatial ability of this subgroup thus probably relates to a younger age, rather than an actual advantage in spatial ability for left-handers in the ‘bias zone’. This would explain why in figure 4*b*,*c*, we see the advantage for left-handers appear in the bias zone. And in participants from the UK and the US (figure 4*f*), a population in which we have found less selection bias [35], we find less effect of handedness on navigation with age than in the global sample. Overall, this analysis supports the view that the divergence in performance between left- and right-handers in older age is due to unreliable participant demographics above 70 years old.

To verify whether handedness had an effect on visuomotor skills, we fit the same multi-level linear model as above, but with the trajectory length of the first two levels as the response variable. The first two levels were tutorial levels where large-scale navigation ability was not required, as the goal was visible from the starting point. Age (*F*_1,422767_ = 5556.50, *p* < 0.001), gender (*F*_1,422767_ = 2323.90, *p* < 0.001) and education (*F*_3,422767_ = 286.15, *p* < 0.001) had a significant effect on performance. On the other hand, handedness did not have a significant effect (*F*_1,422767_ = 1.43, *p* = 0.23). Figure 4*a* shows the trajectory length at the first two levels for each gender and dominant hand. We measured the effect size of handedness on visuomotor skills with Hedges' *g*. Overall, *g* = −0.014, 95% CI = [−0.024, −0.004] (in females *g* = −0.019, 95% CI = [−0.034 −0.003], in males *g* = 0.002, 95% CI = [−0.011, 0.016]), positive values corresponding to better performance in left-handers. As a point of comparison, for gender, *g* = 0.11, 95% CI = [0.11, 0.12], positive values corresponding to better performance in males.

**Figure 5.**
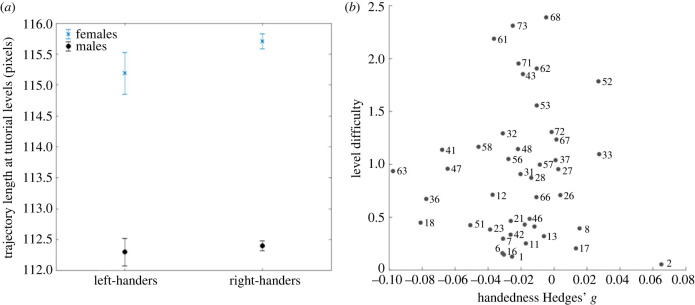
(*a*) Effect of handedness (Hedges' *g* = −0.01) and gender (Hedges' *g* = 0.11) on visuomotor skills. Visuomotor skills are estimated by the trajectory length at the tutorial levels (levels 1 and 2), which did not require any spatial ability. Error bars correspond to 95% confidence intervals. (*b*) Level difficulty as a function of handedness effect size. Effect size has been estimated by Hedges' *g*, with positive values corresponding to an advantage for the right-handers.

We tested whether the effect size of handedness was modulated by task difficulty. We selected a subset of participants who completed all Sea Hero Quest levels (75 levels, 10 626 participants) and computed Hedges' *g* effect size between left-handers and right-handers in all wayfinding levels (*N* = 44, not all Sea Hero Quest levels are wayfinding levels). We did not find a significant correlation between level difficulty and handedness effect size (Pearson's correlation *r* = 0.02, *p* = 0.90, figure 4*b*). As in [56], we used the difference between the median trajectory length and the shortest trajectory length (better optimized) as a proxy for the level difficulty: difficulty = (median(TL) − min(TL))/min(TL), with TL a vector containing the trajectory lengths of all participants at a given level (figure 5).

## 4. Discussion

In this study, we examined the demographic data from 749 037 participants, across 58 countries and navigation performance from 422 772 from 41 countries. We found no reliable evidence supporting a benefit in spatial ability associated with hand preference, but positive evidence for an association with education and age on handedness prevalence. Here, we discuss handedness first in relation to spatial performance and then in terms of demographics.

Our findings challenge previous studies that suggested a significant relationship between an individual's hand preference and their spatial performance in either small-scale [21] or large-scale tasks [7]. There are at least three reasons for this difference in findings: first, previous studies of spatial skill drew conclusions using small sample sizes. As a result, many studies were not designed to adequately address the research question, and those that were may have been susceptible to publication bias against null effects [57]. Our study is the first to employ a large sample size to show a null effect of handedness on spatial ability, an approach that has been successful in other areas of research (e.g. null effects of bilingualism on executive tasks; [58]). Second, in examining spatial navigation, we employed a mobile app with real-world ecological validity [49], while previous studies employed spatial visualization tasks. Third, previous studies drew samples from single cultures, limiting the generalizability of their results. In the present study, we find our null effect to be universal across a broad span of cultures and languages.

Our use of large-population testing generates sufficient power to meaningfully explore the effects of potential moderating factors. Therefore, we examined whether an interaction between handedness and demographic properties impacted the effect of hand preference on wayfinding performance. We found that neither gender, nor age, nor the country, of our participants moderated the effect of handedness on spatial ability. In addition to large-scale navigation performance, we explored whether handedness might have impacted performance through visuomotor ability. However, as measured by our baseline test (distance in the tutorial), we find no evidence for this. We considered whether the effects of handedness only manifested in difficult tasks. But despite previous findings suggesting spatial granularity moderates the effect of handedness on spatial ability (as in [59]), we found the difficulty of our task did not have an effect either (for the effect of environmental difficulty on spatial tasks, see [60,61]).

Demographically, we find an average of 9.94% left-handers overall, consistent with recent estimates (10.6%, [42]). Like previous studies, we find more males report using their left hand compared with women [2,28]. This gender difference is consistent across most countries, with only a few deviating from this pattern. We also find an overall decline in left-handedness with increasing age, as shown previously [62,63]. This finding may be due to a change in attitudes toward left-handedness [39].

Additionally, our results show the ratio of hand preferences varies depending on the country. Only 2.6% of the participants from China were left-handed, a figure over three times smaller than the average for our sample. This finding is consistent with other studies showing that Chinese individuals are less likely than people from other countries to use their left hand. In this context, it has been suggested that attitudes toward left-handers are a proxy for tolerance towards difference more generally [62,64]. While this finding may be partly due to attitudes towards conformity, results may also be influenced by the speed of industrialization in China. In a country with a large influx of students who are the first in their generation to receive education, it may be more cost-effective to centralize resources and teach pupils to use the same hand in classrooms [39]. This is further evidenced by the effect of education. We found that in China, India, Indonesia, Taiwan and Hong Kong, which have the fewest left-handers overall, people who had received tertiary education were less likely to be left-handed when compared with those who had received secondary education or less. By contrast, we found that education had no effect on the rest of the world (as found in [42]). This suggests that the fairly recent urbanization of these countries may play a role in the incidence of left-handedness.

There are several limitations to this study. We use a self-reported measure (dominant writing hand), and in countries with negative attitudes towards left-handedness, participants might be reluctant to report being left-handed. However, a meta-analysis found that self-reporting did not result in a statistical difference in left-handedness prevalence [42]. Another limitation is the selection bias affecting older participants. Further work could examine this selection bias in more detail in an effort to elucidate why male left-handers have better spatial ability after 65 years old. A hypothesis could have been that left-handers used to face increased educational difficulties. However, the absence of association between education and handedness in most countries (except China, India, Indonesia, Taiwan and Hong Kong) does not support this hypothesis. Relatedly, by using a single-item measure of hand preference with icons to illustrate, we may not have captured the full spectrum of an individual's handedness [62], or aspects such as forced switches in handedness during childhood. Nevertheless, a longer questionnaire, such as the full Edinburgh Handedness Inventory, would not have been practical given our experimental paradigm (a mobile gaming application). Another limitation is that, while we draw from a truly international sample, we do not have representation from all countries. Nor can we ignore the cultural variation present within each country we sample and the lack of representation from more traditional societies [62]. Future work along these lines would be valuable, given the relationship between the priority of particular skills (such as fishing over writing) and the cultural significance of handedness [39].

In conclusion, we provide a large sample of participants and countries to explore the impact of handedness on spatial ability. Our results demonstrate that across a large cross-cultural sample, hand preference is not associated with spatial ability. Moreover, our large sample allows us to verify that socio-demographic factors such as age, gender or education do not moderate the relationship between handedness and spatial ability. These results further our understanding of the interplay of handedness and cognition. They also have ramifications in the research design of neuroimaging studies. Our study shows that left-handedness does not confer an increased general spatial ability, so in this respect, we found no support for including handedness in diagnostic screenings for dementia. And within the remit of navigation research, the null effect found in the present work allays the worries concerning the routine practice of excluding left-handers from brain imaging studies.

## Data Availability

A dataset with the preprocessed trajectory lengths and demographic information is available at https://osf.io/xfz8w/?view_only=08e221cfbff4416db02b1b2fda1b9539. The dataset with the full trajectories is available on a dedicated server: https://shqdata.z6.web.core.windows.net/. We also set up a portal where researchers can invite a targeted group of participants to play SHQ and generate data about their spatial navigation capabilities. Those invited to play the game will be sent a unique participant key, generated by the SHQ system according to the criteria and requirements of a specific project. https://seaheroquest.alzheimersresearchuk.org/ Access to the portal will be granted for non-commercial purposes.
